# Supplementary material for: Resolving the full spectrum of human genome variation using Linked-Reads
Source: Genome Res. 2019 Apr;29(4):635–45. doi: 10.1101/gr.234443.118 (PMC6442396; doi:10.1101/gr.234443.118)
Supplement: Supplemental Material [file supp_gr.234443.118_Supplemental_longranger-2.2.zip › longranger-2.2.tar.bz2!/lr2.2-public/lib/stan/cmdstan/stan/doc/api/html/check__matching__sizes_8hpp_source.html]

Stan: src/stan/math/error\_handling/matrix/check\_matching\_sizes.hpp Source File


|  |  |
| --- | --- |
|  | Stan  2.5.0  probability, sampling & optimization |


- Main Page
- Related Pages
- Namespaces
- Classes
- Files

- File List
- File Members

All Classes Namespaces Files Functions Variables Typedefs Enumerations Enumerator Friends Macros Pages

- src
- stan
- math
- error\_handling
- matrix

check\_matching\_sizes.hpp

Go to the documentation of this file.

1 #ifndef STAN\_\_MATH\_\_ERROR\_HANDLING\_\_MATRIX\_\_CHECK\_MATCHING\_SIZES\_HPP

2 #define STAN\_\_MATH\_\_ERROR\_HANDLING\_\_MATRIX\_\_CHECK\_MATCHING\_SIZES\_HPP

3

4 #include <stan/meta/traits.hpp>

5 #include <stan/math/error\_handling/dom\_err.hpp>

6 #include <string>

7 #include <typeinfo>

8 #include <stan/math/error\_handling/matrix/check\_size\_match.hpp>

9

10 namespace stan {

11  namespace math {

12

13  // NOTE: this will not throw if y1 or y2 contains nan values.

14  template <typename T\_y1, typename T\_y2, typename T\_result>

15  inline bool check\_matching\_sizes(const char\* function,

16  const T\_y1& y1,

17  const char\* name1,

18  const T\_y2& y2,

19  const char\* name2,

20  T\_result\* result) {

21  stan::math::check\_size\_match(function,y1.size(), "size of y1",

22  y2.size(), "size of y2",result);

23  return true;

24  }

25

26  }

27 }

28 #endif

dom\_err.hpp

check\_size\_match.hpp

stan::math::check\_size\_match

bool check\_size\_match(const char \*function, T\_size1 i, const char \*name\_i, T\_size2 j, const char \*name\_j, T\_result \*result)

**Definition:** check\_size\_match.hpp:13

traits.hpp

stan::math::check\_matching\_sizes

bool check\_matching\_sizes(const char \*function, const T\_y1 &y1, const char \*name1, const T\_y2 &y2, const char \*name2, T\_result \*result)

**Definition:** check\_matching\_sizes.hpp:15


---

[ Stan Home Page ]

*© 2011–2014,
Stan Development Team.*
